# *O*-Carboxymethyl Chitosan Supported Heterogeneous Palladium and Ni Catalysts for Heck Reaction

**DOI:** 10.3390/molecules22010150

**Published:** 2017-01-18

**Authors:** Dongjun Lv, Mingjie Zhang

**Affiliations:** 1 Department of Chemistry, School of Science, Tianjin University, Tianjin 300354, China; 2 Shandong YuHong New Pigment Co., Ltd., Dezhou 253000, China

**Keywords:** *O*-carboxymethyl chitosan, biopolymer-supported catalyst, Pd-OCMCS, Ni-OCMCS, heck reaction

## Abstract

Two polymer catalysts (Pd-OCMCS and Ni-OCMCS) with good reusability were synthesized by coordinating Pd and Ni onto *O*-carboxymethyl chitosan (OCMCS). The chemical structure and thermal stability of prepared catalysts were determined by Fourier transform infrared (FT-IR) spectra, Energy Dispersive Spectrometer (EDS)analysis, X-ray diffraction (XRD), and thermogravimetric analyzer (TG-DTG), and the analysis results showed that the Pd and Ni ions coordinated onto the OCMCS and formed a ligand with the –COOH group, amino groups, and –OH group on the OCMCS, and the EDS and Inductively Coupled Plasma Optical Emission Spectrometry (ICP-OES) analysis results showed that the loading amounts of Pd and Ni were approximately 8.3% and 8.9%, respectively. In the Heck reaction between aryl halides and *n*-butyl acrylate catalyzed by the prepared catalyst, the test results showed that the product yield followed the order of aryl iodide > aryl bromide > aryl chloride. Additionally, the product yield for the aryl iodide and aryl bromide could reach up to 99% and 96%, respectively. Moreover, the electron-withdrawing and electron-donating property of the group on the aryl also affected the product yield, and the product yield for aryl halides with electron-withdrawing group *p*-NO_2_, *p*-CH_3_CO, and *p*-CHO was higher than that with electron-donating group *p*-CH_3_.

## 1. Introduction

Heck cross-coupling is an important method for carbon–carbon bond synthesis, and has greatly attracted more and more attention for its wide application in the synthesis of dyes, medicines, natural products, pesticides, perfumes, cinnamic acid derivatives, and new polymer materials [1,2,3,4,5]. The palladium-catalyzed Mizoroki–Heck cross-coupling reaction represents one of the most valuable methods for carbon–carbon bond formation in organic synthesis [6,7]. However, its usage is limited in homogeneous reaction systems due to the difficulties in the separation of the product and catalyst from the reaction mixture and the recycling of catalyst. In order to overcome the aforementioned drawbacks, the heterogenization of catalysts has been studied extensively [4,8,9].

Bio-based polymers have attracted worldwide attention because of their potential biomedical applications, and have served as catalyst carriers in chemical reactions [10,11,12]. Chitosan and its derivatives have highly efficient polymer ligands due to their good metal binding properties [13,14]. Khaled D. Khalil et al. [15] synthesized chitosan-grafted-poly(4-vinylpyridine) beads for Michael additions, and it was a recyclable, environmentally friendly biocatalyst. Hardy et al. [16] synthesized chitosan Schiff bases, prepared a Pd(II) complex, and investigated its catalytic activity in Heck and Suzuki reactions. Talat Baran et al. [17] prepared Cu(II) and Pd(II) complexes with water soluble *O*-carboxymethyl chitosan (OCMCS) Schiff bases, and they assumed that these biopolymer-based Cu(II) and Pd(II) complexes could be widely used in different fields, such as catalytic activity improvement, DNA cleaving, and antibacterial and anticancer research.

In the C–C bond-forming reactions, palladium-based catalysts are the optimal choice due to their high catalytic activity. However, the application of palladium-based catalysts is limited because of their high cost, and people’s attention has turned to nickel-based catalysts with low cost. In some coupling reactions cases, nickel-based catalysts showed higher catalytic activity than palladium-based catalysts [18,19]. Currently, functional chitosan-based materials containing nickel have aroused high interest in the fields of organic catalysis [20]. A.S. Portnyagin et al. [21] investigated Ni^2+^ complexes with chitosan and its *N*-heterocyclic derivatives with density functional theory (DFT), and they found that the nitrogen atoms in chitosan and its *N*-heterocyclic derivatives played a governing role in Ni^2+^ binding. Antony et al. [22] fabricated a new class of bidentate (N, O) Schiff base ligand (L) derived from the functional biopolymer (chitosan) and 1,2-diphenylethanedione in a 1:1 mole ratio, and they utilized the ligand to synthesize new first-row transition metal complexes of Cu(II), Co(II), and Ni(II).

Herein, we prepared two polymer catalysts by coordinating Pd and Ni onto *O*-carboxymethyl chitosan (OCMCS). The chemical structure and thermal stability of the prepared catalysts were determined by Fourier transform infrared (FT-IR) spectra, X-ray diffraction (XRD), Energy Dispersive Spectrometer (EDS) analysis, and thermogravimetric analyzer (TG-DTG), and the catalytic activity of the prepared catalyst in Heck reaction between aryl halides and *n*-butyl acrylate was tested.

## 2. Results

### 2.1. FT-IR Spectra

**Figure 1:**
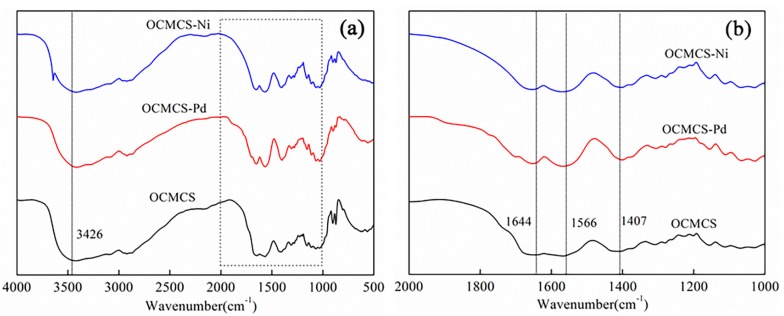
FT-IR spectra of *O*-carboxymethyl chitosan (OCMCS, black line), OCMCS-Pd (red line), OCMCS-Ni (blue line) in the wavenumber range of 500–4000 cm^−1^ (**a**); and the wavenumber range of 1000–2000 cm^−1^ (**b**).

FT-IR spectra were used to determine which functional groups in OCMCS coordinated with the two metals, palladium and nickel, and the results are shown in Figure 1.

In the curve of OCMCS, the characteristic adsorption peaks at 1407 and 1644 cm^−1^ correspond to the symmetric and asymmetrical stretching bands of carboxylate anion, and the second adsorption peak (1644 cm^−1^) overlaps with the N–H bend. In order to investigate the interaction between Pd and Ni ions and OCMCS, infrared spectroscopy in the wavenumber range 1000–2000 cm^−1^ was observed on a large scale, and the results are shown in Figure 1b. It could be seen that in the spectrum of OCMCS-Pd and OCMCS-Ni, the asymmetric C=O stretching vibration peak shifted to 1654 and 1650 cm^−1^, respectively, and C–O symmetric stretching absorption peak were shifted to 1401 and 1403 cm^−1^, respectively. Thus, it could be concluded that in OCMCS-Pd and OCMCS-Ni, the Pd and Ni ions bind to the carboxylate groups of OCMCS.

Similarly, the characteristic peaks at 1566 cm^−1^ belonged to the stretching vibration absorption peak of N–H in the –NH_2_ group shifted to 1561 cm^−1^ in the curve of OCMCS-Pd and 1568 cm^−1^ in the curve of OCMCS-Ni, and the characteristic peaks at 3426 cm^−1^ belonged to the stretching vibration absorption peak of O–H in the –OH group shifted to 3415 cm^−1^ in the curve of OCMCS-Pd and 3418 cm^−1^ in the curve of OCMCS-Ni. As stated above, in the prepared OCMCS-Pd and OCMCS-Ni catalysts, the palladium and nickel ions also bind to the –NH_2_ and –OH groups in OCMCS.

### 2.2. Thermal Analysis

**Figure 2:**
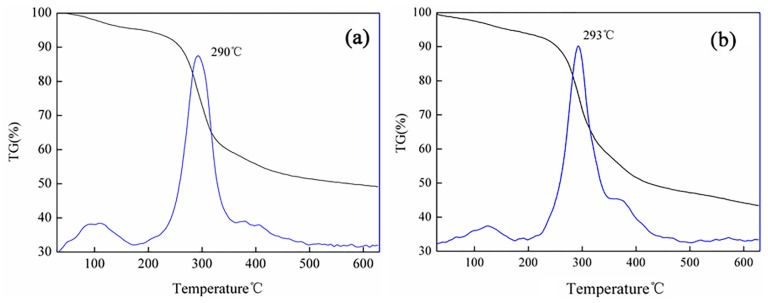
TG/DTG spectra of (**a**) OCMCS-Pd and (**b**) OCMCS-Ni.

Thermal stability of the prepared OCMCS-Pd and OCMCS-Ni catalyst were tested by TG–DTG measurements under Ar atmosphere, and the results are shown in Figure 2. It can be seen from the DTG curves of OCMCS-Pd and OCMCS-Ni that their exothermic peaks were recorded at 290 °C and 293 °C, respectively, suggesting that the prepared catalyst showed a high thermally stability, and that they could be utilized in the C–C coupling reactions [17].

### 2.3. XRD Analysis

**Figure 3:**
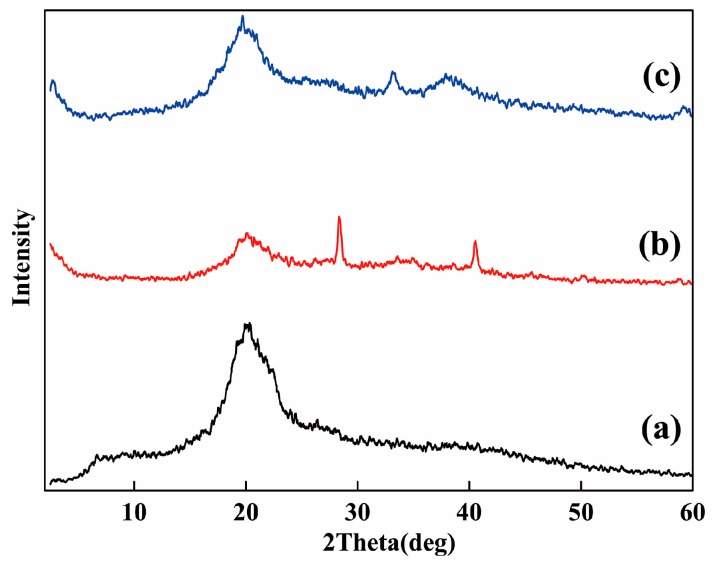
Powder X-ray diffraction diagrams of (**a**) OCMCS; (**b**) OCMCS-Pd; and (**c**) OCMCS-Ni.

The prepared catalyst and OCMCS were characterized by X-ray diffraction (XRD), and the results are shown in Figure 3. An obvious diffraction peak at 2θ = 20.1° was observed in the OCMCS pattern. By comparison, it could be seen that, in the prepared catalyst, the diffraction peak of OCMCS showed a decrease in its diffraction intensity as well as a peak broadening phenomenon, caused by the formation of coordinated ligands between OCMCS and palladium/nickel ions mentioned in the FT-IR analysis [22,23,24]. In addition, in the XRD patterns of the prepared catalyst, new diffraction peaks at 2θ = 28.4°, 40.5° in the pattern of Pd-OCMCS (Figure 3b) and new diffraction peaks at 2θ = 33.2°, 38.1° in the pattern of Ni-OCMCS (Figure 3c) were observed, implying that the Pd and Ni ions exist in a crystalline state on the OCMCS [25].

Moreover, according to the following equation, it could be calculated that the OCMCS-Ni catalyst showed higher crystallinity values than the OCMCS-Pd catalyst [26].

Crystalline index (%) = [(I_110_ − I_am_)/I_110_] × 100

In which the I_110_ was the maximum intensity at 20° and I_am_ was the intensity of amorphous diffraction at 16°.

### 2.4. SEM–EDS Analysis

**Figure 4:**
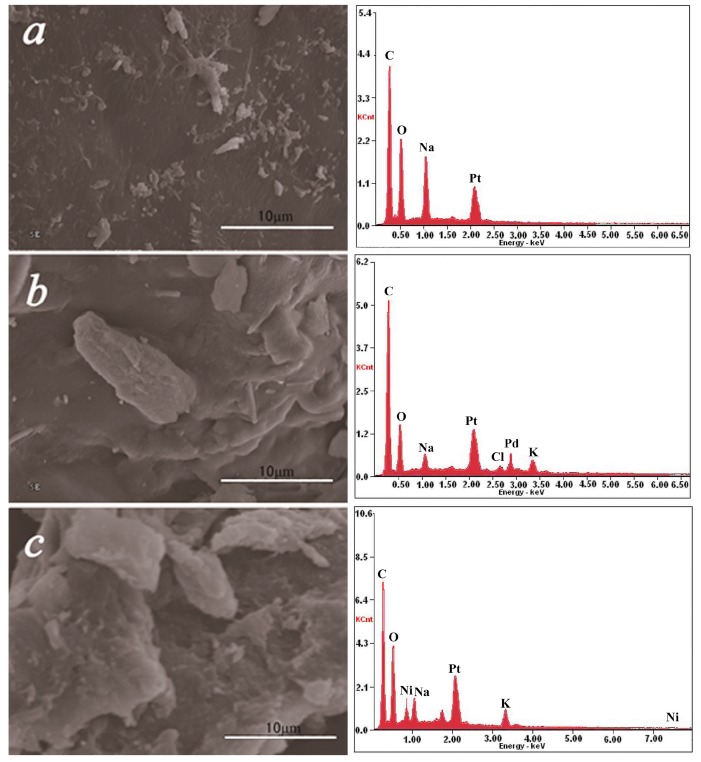
Scanning electron microscopy pictures and SEM/EDS spectra of (**a**) OCMCS; (**b**) OCMCS-Pd; (**c**) OCMCS-Ni.

The morphology of OCMCS and prepared catalyst were characterized by SEM, and the results are shown in Figure 4. It could be found that the morphologies and particle size of prepared OCMCS-Pd and OCMCS-Ni catalysts were completely different from those of OCMCS, and the prepared catalysts had more regular morphologies. As mentioned in the FT-IR analysis results, the Pd and Ni ions bind to OCMCS through the –COOH, –NH_2_, and –OH groups, which resulted in a change of the morphology of OCMCS [27]. The EDS spectra were recorded in order to further confirm the presence of the Pd and Ni ions in the prepared catalyst, and it was obvious that the presence of the Pd element in OCMCS-Pd and the Ni element in OCMCS-Ni was determined. Additionally, their loading amount was determined using ICP-OES spectra, and the results showed that the loading amounts of Pd and Ni were approximately 8.3% and 8.9%, respectively.

### 2.5. Catalytic Studies

#### 2.5.1. Heck Cross-Coupling Reactions

The catalytic activities of the prepared OCMCS-Pd and OCMCS-Ni catalysts were tested by the Heck cross-coupling reactions between various substituted aryl halides and *n*-butyl acrylate, and the results are shown in Table 1 and Table 2. For aryl halides in which the ring was substituted by different halogens, the activity of the halides in reactions catalyzed by OCMCS-Pd and OCMCS-Ni followed the order of iodide > bromide > chloride.

In reactions with aryl iodides and aryl bromides, product yields of up to 99% and 96%, respectively, were obtained (Table 1, entries 3 and 7). However, the highest yield obtained in reactions of aryl chlorides was only 51% (Table 1, entry 8).

For the same aryl halides, the OCMCS-Pd showed a higher catalytic activity than OCMCS-Ni. As shown in Table 1 and Table 2, in reactions of aryl chlorides with catalyst OCMCS-Pd, product yields of up to 51% could be obtained (Table 1, entry 8). However, in the presence of catalyst OCMCS-Ni, the same aryl chlorides did not react at all under the same reaction conditions (Table 2, entries 8 and 9). It was also found that the electronic properties of aryl halide substituents influenced the yields of the products. It may be seen from Table 1 and Table 2 that product yields were higher when the aryl halides contained electron-withdrawing substituents (e.g., *p*-NO_2_, *p*-CH_3_CO and *p*-CHO) than when they contain electron-donating substituents (e.g., *p*-CH_3_); i.e., the catalytic activity was higher when aryl halides containing electron-withdrawing substituents were used. It could be seen from Table 1 (entries 8 and 9) that the withdrawing group *p*-NO_2_ improved the product yield of chlorobenzene catalyzed by OCMCS-Pd by 45%.

#### 2.5.2. Reusability of Catalysts

**Figure 5:**
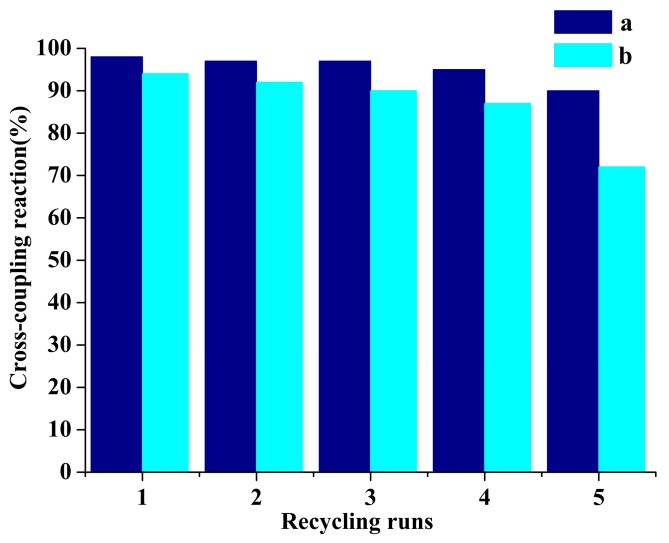
Dependence of the cross-coupling yield on the recycling run with the recovered (**a**) OCMCS-Pd and (**b**) OCMCS-Ni catalysts.

After Heck reaction of iodobenzene and *n*-butyl acrylate, the prepared catalysts were recycled by simple filtration, washing with ethanol, and oven drying, and then the leaking amount of Pd and Ni ions in the filtrate and the catalytic activity of recycled catalyst were tested to evaluate the reusability of prepared catalysts, and the results are shown in Figure 5. Firstly, the results of Inductively Coupled Plasma Optical Emission Spectrometry (ICP-OES) analysis showed that the leaking amount of Pd and Ni ions in the filtrate containing the products were only 0.021 and 0.052 mg/kg, respectively, suggesting that the Pd and Ni were well immobilized on the OCMCS. Secondly, it could be seen from Figure 5 that after recycling the OCMCS-Pd and OCMCS-Ni five times, they still showed high cross-coupling reaction activity, and the cross-coupling reactions for OCMCS-Pd and OCMCS-Ni were still over 90% and 72%, suggesting that the prepared catalyst showed good reusability.

## 3. Discussion

In this paper, two polymer catalysts were synthesized by coordinating Pd and Ni onto the *O*-carboxymethyl chitosan (OCMCS). The FT-IR spectral analysis results showed that the Pd and Ni ions coordinated to OCMCS via the carboxyl, amino, and hydroxyl groups [14]. The thermal analysis results showed that the links formed between carboxymethyl chitosan and the metals in the prepared catalysts have good thermal stability, and hence the catalysts could be utilized in the C–C coupling reactions. The EDS analysis and ICP-OES analysis results showed that the loading amount of Pd and Ni on the prepared catalysts were approximately 8.3% and 8.9%, respectively.

The test results of the Heck reaction between aryl halides and *n*-butyl acrylate catalyzed by the prepared catalyst showed that the product yield was affected by the halogen species and the electron-withdrawing and electron-donating property of groups on the aryl. The product yield for the aryl iodide and aryl bromide could reach up to 99% and 96%, respectively, and the product yields for the aryl halides with electron-withdrawing groups *p*-NO_2_, *p*-CH_3_CO, and *p*-CHO were higher than that with electron-donating group *p*-CH_3_. After reaction, the ICP-OES analysis results showed that the leaking amount of Pd and Ni ions during the reaction was only 0.021 and 0.052 mg/kg. Additionally, the prepared OCMCS-Pd and OCMCS-Ni catalysts still showed 90% and 72% reaction activity after five times recycling, suggesting that the prepared catalyst possessed good reusability.

## 4. Materials and Methods

### 4.1. Materials

Aryl halides were obtained from Shanghai Yuanye Bio-Technology Co., Ltd. (Shanghai, China). Palladium chloride, nickelous acetate, *N*,*N*-dimethylformamide, and butyl acrylate were obtained from Shanghai Aladdin Bio-Chem technology Co., Ltd. (Shanghai, China). *O*-Carboxymethyl chitosan (OCMCS) was purchased from Shanghai Dibo Chemical Technology Co., Ltd. (Shanghai, China).

### 4.2. Physical Measurements

The FT-IR spectra of samples were performed on a FT-IR spectrophotometer (Nicolet I5 America). The thermal stability of the prepared catalyst was analyzed by a thermogravimetric analyzer (NETZSCH STA 449F3, Selb, Germany) under an argon atmosphere at a heating rate of 10 °C·min^−1^. The level of crystallinity of the prepared catalysts and OCMCS was analyzed by X-ray diffraction (XRD, MiniFlex 600, Rigaku, Tokyo, Japan) under a 2θ scan angle from 2.5° to 60°. The surface morphologies of the samples were examined using field emission scanning electron microscope (S-4800, Hitachi, Tokyo, Japan), and their EDS spectra were determined by EDAX-Metek. In addition, the loading amounts of Pd and Ni on OCMCS were determined with a plasma optical emission spectrometer (Optima 8000, PerkinElmer, Waltham, MA, USA).

### 4.3. Synthesis of OCMCS-Pd and OCMCS-Ni

Firstly, the PdCl_2_ and C_4_H_6_NiO_4_ aqueous solutions were prepared by the following procedure: the PdCl_2_ (0.35 g) and HCl (0.5 g) were dissolved in 10 mL water and stirred for 1 h at room temperature to obtain a complete aqueous solution. Then, the nickel acetate tetrahydrate (1.24 g) was dissolved in 30 mL water.

Secondly, the OCMCS-Pd catalyst was synthesized. At room temperature, the *O*-carboxymethyl chitosan (OCMCS, 1.97 g) was added into a KOH aqueous solution (0.4 g KOH, 40 mL water) and stirred at 60 °C for 1 h. Then, the prepared water soluble PdCl_2_ aqueous solution was added over 30 min to react with OCMCS for 12 h, and the reaction temperature was 60 °C. Then, the reaction solution was poured into a round-bottomed flask, and the water was removed with a rotary evaporator to obtain the concentrated solution. After cooling to room temperature, the ethanol was added into the concentrated solution to give a precipitate. The mixture was filtered, washed with ethanol, and dried at 60 °C in an oven to give the Pd-OCMCS catalyst.

The OCMCS-Ni catalyst was synthesized according to the above procedure, but 2.63 g of OCMCS and 50 g of water were used instead.

### 4.4. General Procedure for the Heck Reaction

In a typical reaction, the aryl halides (1 mmol), *n*-butyl acrylate (1.1 mmol), Et_3_N (1.5 mmol), and the prepared catalyst were added into the reaction vessel charged with *N*,*N*-dimethylformamide (DMF, 5 mL), and then the reaction mixture was heated to 140 °C for 12 h. After the completion of the reaction, the mixture was cooled down to room temperature and filtered. The filtered cake was washed with ethanol and vacuum dried for reuse. The filtrate was analyzed by a gas chromatograph-mass spectrometer (GC-MS, QP2010Plus, Shimadzu, Kyoto, Japan) to determine the yield of biaryl compound.

## Figures and Tables

**Table 1:**
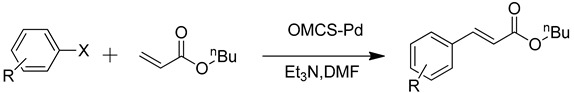
Heck reaction of aryl halides and *n*-butyl acrylate catalyzed by OCMCS-Pd ^a^.

Entry	ArX	Product	Yield ^b^ (%)
1	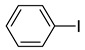	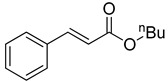	98
2	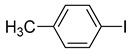	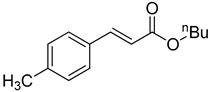	94
3	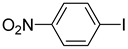	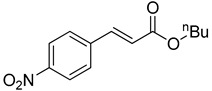	99
4	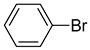	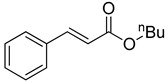	89
5	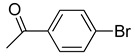	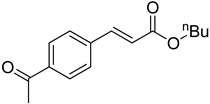	94
6	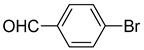	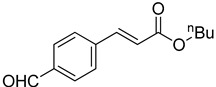	93
7	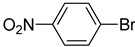	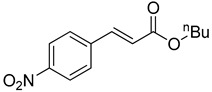	96
8	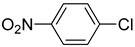	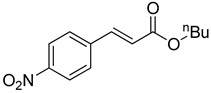	51
9	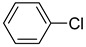	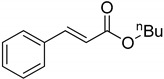	6

^a^ Reaction conditions: aryl halides (1.0 mmol), *n*-butyl acrylate (1.1 mmol); OCMCS-Pd (0.02 mmol Pd), *N*,*N*-dimethylformamide (DMF) (5.0 mL) at 140 °C for 12.0 h; ^b^ The reaction yields were determined by GC-MS analysis of the crude reaction product.

**Table 2:**
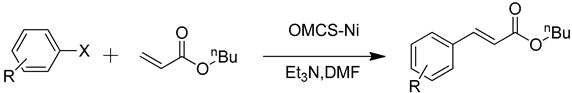
Heck reaction of aryl halides and *n*-butyl acrylate catalyzed by OCMCS-Ni ^a^.

Entry	ArX	Product	Yield ^b^/%
1	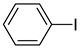	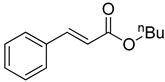	94
2	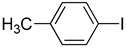	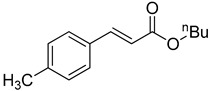	86
3	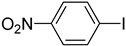	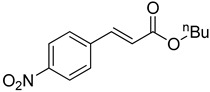	88
4	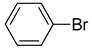	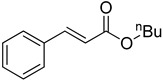	67
5	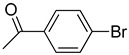	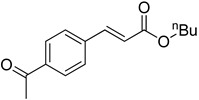	79
6	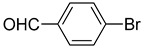	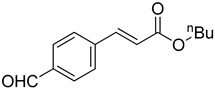	72
7	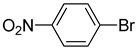	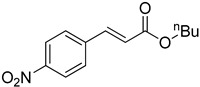	87
8	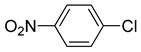	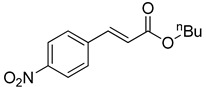	-
9	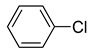	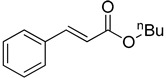	-

^a^ Reaction conditions: aryl halides (1.0 mmol), *n*-butyl acrylate (1.1 mmol); OCMCS-Ni (0.04 mmol Ni), DMF (5.0 mL) at 140 °C for 12.0 h; ^b^ The reaction yields were determined by GC-MS analysis of the crude reaction product.
